# Beri-Beri

**Published:** 1914-05

**Authors:** Paul G. Woolley

**Affiliations:** University of Cincinnati


### Beri-Beri.

BY PAUL G. WOOLLEY, M. D., UNIVERSITY OF CINCINNATI.

The causes which have been assigned for the symptom-complex we call beri-beri are legion. Almost everything under the sun, and perhaps some above it, have been seriously discussed as etiologic factors. It has been ascribed to intestinal parasites, protozoa and bacteria, to occupation and to diet, and it has been suspected of being collateral result of malarial infection and other infectious diseases, and of scurvy.

The most important theories of etiologic significance are the following:

(1) That the disease is the expression of the lack of some normal constituent of food, i. e., that it is due to nitrogen or phosphorus starvation.

(2) That it is the result of the presence of arsenic in the food.

(3) That it is the result of intoxication with products of decomposition of food outside the body, i. e., that it is a form of food poisoning.

(4) That it is a soil or place disease.

(5) That it is the result of intoxication by bacterial products in the body, especially in the gastrointestinal tract (Wright), or in the upper respiratory tract (Durham).

(6) That it is the result of infection.

(7) That it may be the result of any one or several of the above factors.

The important ones among these are the first, third and seventh. These I shall discuss briefly. The others are mentioned for the sake of completeness.

The *nitrogen starvation hypothesis* originally was based upon the fact that beri-beri is most frequent among rice-eating peoples and in general among those who subsist almost exclusively upon food that is deficient in nitrogen. To test the validity of the theory, an experiment was made in the Japanese navy, which up to 1883 had shown a morbidity of 25 per cent. In 1884 the diet was changed, less rice was issued, and other articles of diet richer in nitrogen were substituted, with the result that the disease was almost abolished, and has remained a comparatively unimportant item in the sick list ever since. But the validity of the hypothesis was not proved by these dietary changes, for the reason that hygienic changes were instituted at the same time. Furthermore, inconclusive experiments following the same lines were made in the Malay States. Then, too, in other parts of the world where diet poor in nitrogen is used, beri-beri is of but limited extent. It also occurs in individuals who use a diet rich in nitrogen, as, for instance, among Norwegians and Swedish sailors. In certain of the Philippine Islands where rice was not the main diet beri-beri occurred (Heiser).

Frazer and Stanton brought evidence to support the suggestion of Braddon that a casual relationship exists between beri-beri and the use of white rice, and to account for the observation that persons who were in the habit of using rice that was parboiled before husking and those who used the primitive method of husking and winnowing were less subject to the disease than those who used rice which was not steamed before husking and those who used polished rice. It had been suggested that the parboiling was effective in destroying organisms that were present upon the grains (Braddon), and that the native methods of pounding and winnowing the grain left a sufficient amount of the pericarp and that in this way a substance that had antidotal effects upon a poison commonly present in white rice. It is unnecessary to discuss the presence of such a poison. It has not been proved to exist. Nevertheless there has been, in certain localities, an apparent relation between the use of polished milled rice and the beri-beri incidence. It is of some interest that rice which has been supposed to be the casual factor in the production of beri-beri also has been able to produce an analgous disease in fowls (*polyneuritis gallinarum*), while parboiled rice does not produce such results, though after extraction with alcohol and subsequent careful drying, it will produce the same effect. The observed facts lead to analyses of the various sorts of rice, analyses that resulted in the discovery that certain rice, especially the "White Siam," was markedly deficient in fats and somewhat low in ash. It was also found that the phosphorus content of rice which produced polyneuritis in fowls was far below (about 50 per cent) that of parboiled rice, while the polishings collected at a rice mill contain a large amount of phosphorus. It was estimated that one fowl required 60 grammes of parboiled rice to maintain phosphorus equilibrium, and that, using polished rice, an additional 3.5 grammes of polishings was necessary. It appears then from recent work that a diet poor in phosphorus is able to produce in fowls a disease analogous to beri-beri, and that the lack of phosphorus in polished rice is the result of the milling methods used in polishing the grains, since the greater part of the phosphorus of the rice is held in the pericarp. Parboiling renders the removal of the pericarp more difficult.

Notwithstanding the fact that many investigations have supported, in a general way, the phosphorus doctrine, there remains the possibility that something beside the mere phosphorus content of the food it at fault. Aron, and Aron and Hocson believed, upon the basis of their experimental evidence, that both phosphorus and nitrogen play a part, and de Haan that something beside phosphorus is at fault. He thinks it is neither salts nor nucleins. Perhaps it is the lipoids. Shibayama does not think that overmilled white rice is the sole cause of beri-beri. His experiments with miners seem to show that workers who received fresh unmilled rice were more susceptible to beri-beri than those fed exclusively with milled rice. Shibayama comes to the conclusion that a monotonous one-sided diet predisposes to beri-beri, and *only* predisposes to it. The immediate cause he does not specifically suggest, but he calls attention to the interesting fact that in beri-beri districts many persons become affected with beri-beri during convalescence from typhoid fever, a thing which does not happen in other districts. This fact, Shibayama believes, points to the existence of a still unknown beri-beri organism. He, however, apparently fails to take rescrudescence into consideration.

The conception of a badly balanced diet as a predisposing cause seems a not unreasonable one in view of the fact that limited diets have a harmful effect on many animals as well as upon human beings. Wherry, Hunt and others called attention to the fact that the resistance of animals to infection is lowered by such diets. Wherry found, for instance, that when guinea pigs had been fed upon an exclusive diet of barley and water, they became scorbutic and that then they showed a greater degree of hemorrhagic extravasation when inoculated with plague than was the rule with other guinea pigs fed with a more normal diet. Hunt said, that "diet has a marked effect upon the resistance of animals to certain poisons," and further, "that foods such as enter largely into the daily diet of man have most pronounced effects upon the resistance of animals to several poisons; they produce changes in metabolism which are not readily detectable by methods ordinarily used in metabolism studies. The ease and rapidity with which certain changes of function are caused by diet are in striking contrast with the essentially negative results obtained by the chemical analyses of animals fed on different diets." Such a lowering of resistance plus the additional specific effects upon the nervous system caused by lack of certain necessary salts of phosphoric acid might well be conceived to be the cause of the symptom complex of kakke.

Infected food, more particualrly decayed fish and old spoiled rice, have been under suspicion of producing beri-beri.

Fish, however, has been excluded from the probable general causes, because, during epidemics in which food stuffs have been under control, as for instance, in a Kuala Lampur epidemic, no fish was used.

Rice, on the other hand, is still under suspicion. Braddon's theory is that the husk of the rice grains may contain a fungus which is able to penetrate the bruised and decorticated grain, and that only certain crops of rice are infected with the fungus and only, perhaps, the crops from certain places. In support of this is the fact that the Tamils steam or boil their rice before husking, while the Chinese do not, and that outbreaks of the disease may be stopped by changing the rice. In northern Siam epidemics occur during high water at times when fresh rice is not to be had, and when all other vegetable diet is excluded because of the general conditions. During these times the Laos subsist entirely upon the rice of previous seasons, and this in seasons when the conditions of temperature and moisture are most favorable for the growth of fungi in the rice of the storehouses. With opportunity for obtaining new rice or other food the epidemics cease. One might refer such epidemics to a combination of nitrogen starvation plus intoxication from spoiled rice.

There is the possibility that a combination of two or more of the above mentioned factors may account for the disease, a possibility that seems most probable, inasmuch as the pathologic changes are not pathognomonic and merely indicate an intoxication of mild or severe character which results in a peripheral neuritis associated with myocardial, vascular, and voluntary muscular changes. In the foregoing paragraphs this possibility has been emphasized, and it seems to be one which has attracted the attention of very many of the modern writers. Aron and Hocson emphasize this when they say that "It is highly probable that living for an extended period on a one-sided, almost exclusively vegetable diet, which is characterized by its poverty in phosphorus and protein, may result in beri-beri." That is to say, not merely does the disease result from phosphorus but also nitrogen starvation. But it still seems very probable, as Shibayama and others assert, that these dietary deficiences are merely predisposing and not immediately causative. Brooks mentioned the fact that beri-beri was observed in the Straits Settlements ten years before steam-milled rice was in use; Kilbourne stated that it was present in the Philippines before the use of polished rice was in vogue, and Highet made a similar remark for the occurrence of beri-beri in Siam. I know personally that beri-beri epidemics have appeared among the Laos of northern Siam who used no other rice than that which was pounded, and not milled. All of these, and other facts, point to the conclusion that some other factor than lack of phosphorus and nitrogen concerns the appearance of the disease, and this, taken together with the pathologic facts, point to a lack of unity in this missing factor. Barbezieux goes to another extreme and calls in an hypothetical neuro-arthritic diathesis. But he also says that beri-beri is a syndrome which depends upon nutritional disturbances. Aron and Hocson suspect that some unknown factor exists that causes the "wet" form, a suspicion which has been supported by Vedder and Clark.

The facts that there is generally some relation between the use of rice and the beri-beri incidence; that the use of undermilled rice seems to decrease the incidence; that when polished rice is combined with either the millings, or with extracts of the millings, the incidence is less; that the use of millings, or extracts of millings, serve usefully in the treatment of beri-beri —all these facts have turned research to the discovery of the vital element in the rice, in the absence of which beri-beri appears. Whether the active substance is a phosphorus compound one or not; whether it is a salt or a lipoid has held the attention of many workers, among whom may be mentioned Frazer and Stanton, Chamberlain and Vedder, Schaumann, Shiga, Strong and Crowell, Funk and Suzüki.—*American Journal of Tropical Diseases and Preventive Medicine.*

